# Methods, Indicators, and End-User Involvement in the Evaluation of Digital Health Interventions for the Public: Scoping Review

**DOI:** 10.2196/55714

**Published:** 2024-05-31

**Authors:** Vera Weirauch, Clarissa Soehnchen, Anja Burmann, Sven Meister

**Affiliations:** 1 Health Informatics Faculty of Health, School of Medicine Witten/Herdecke University Witten Germany; 2 Fraunhofer Institute for Software and Systems Engineering Dortmund Germany

**Keywords:** digital health, digital health intervention, public end user, evaluation methods, evaluation criteria, end-user involvement, scoping review

## Abstract

**Background:**

Digital health interventions (DHIs) have the potential to enable public end users, such as citizens and patients, to manage and improve their health. Although the number of available DHIs is increasing, examples of successfully established DHIs in public health systems are limited. To counteract the nonuse of DHIs, they should be comprehensively evaluated while integrating end users. Unfortunately, there is a wide variability and heterogeneity according to the approaches of evaluation, which creates a methodological challenge.

**Objective:**

This scoping review aims to provide an overview of the current established processes for evaluating DHIs, including methods, indicators, and end-user involvement. The review is not limited to a specific medical field or type of DHI but offers a holistic overview.

**Methods:**

This scoping review was conducted following the JBI methodology for scoping reviews based on the framework by Arksey & O’Malley and complies with the PRISMA-ScR (Preferred Reporting Items for Systematic Reviews and Meta-Analyses extension for Scoping Reviews) guidelines. Three scientific databases (PubMed, Scopus, and Science Direct) were searched in April 2023. English and German studies between 2008 and 2023 were considered when evaluating DHIs that explicitly address public end users. The process of study selection was carried out by several researchers to avoid reviewer bias.

**Results:**

The search strategy identified 9618 publications, of which 160 were included. Among these included articles, 200 evaluations were derived and analyzed. The results showed that there is neither a consensus on the methods to evaluate DHIs nor a commonly agreed definition or usage of the evaluated indicators, which results in a broad variety of evaluation practices. This aligns with observations of the existing literature. It was found that there is a lack of references to existing frameworks for the evaluation of DHIs. The majority of the included studies referred to user-centered approaches and involved end users in the evaluation process. As assistance for people developing and evaluating DHIs and as a basis for thinking about appropriate ways to evaluate DHIs, a results matrix was created where the findings were combined per DHI cluster. Additionally, general recommendations for the evaluators of DHIs were formulated.

**Conclusions:**

The findings of this scoping review offer a holistic overview of the variety and heterogeneity according to the approaches of evaluation of DHIs for public end users. Evaluators of these DHIs should be encouraged to reference established frameworks or measurements for justification. This would ease the transferability of the results among similar evaluation studies within the digital health sector, thereby enhancing the coherence and comparability of research in this area.

## Introduction

### Background

The potential of digital health interventions (DHIs) to improve care processes is widely recognized [1,2]. Particularly for public end users, DHIs have the potential to enable them to manage and improve their health by promoting health, supporting behavior change, personalizing health care delivery, and giving them the chance to individually organize health care [1-6]. Consequently, the development of DHIs is increasing, and a growing number of them are available in the market [3,4]. Nevertheless, examples of successfully established DHIs in public health systems are limited [7], and several challenges exist in this rapidly growing field, for example, methodological challenges [1,6,8,9].

Before elaborating on the challenges, it is useful to consider the term DHI from a definitional point of view. There are various terms for digital technologies in the health sector, such as eHealth, mobile health (mHealth), digital health services, and DHIs. To avoid conceptual ambiguity, this paper adopts the definitional approach of the World Health Organization (WHO), defining a DHI as “a discrete functionality of the digital technology to achieve health sector objectives” [10]. Following this definition, the growing field of digital public health interventions (DPHIs) can be regarded as a distinct subset of DHIs. DPHIs primarily focus on improving health and well-being at the population level, rather than at the individual level [11-13]. For this study, the definition of the key term “evaluation” has also been adopted according to the WHO, which has defined evaluation as “The systematic and objective assessment of an ongoing or completed intervention, with the aim of determining the fulfillment of objectives, efficiency, effectiveness, impact, and sustainability” [14]. Additionally, it is important to specify that in the context of this scoping review, “public end users” are defined as individuals, such as citizens and patients, who directly interact with digital health tools or services. Unlike health care professionals or caregivers, who may use DHIs as part of their job, public end users engage with these tools to meet their personal health needs. They are not limited to any specific patient group or demographic.

#### Status Quo on the Evaluation of DHIs

Overall, DHIs are characterized as complex interventions. This complexity is partly due to their interdisciplinary and multisectoral nature, involving a diverse mix of stakeholders, including patients, various health professionals, relatives, policymakers, and health insurers [1,3,8,15]. Additionally, DHIs consist of multiple interdependent components, both technical and nontechnical [4,8,15], and serve multiple aims, such as providing information, improving communication, facilitating data sharing, and enabling monitoring [1]. The value and impact of DHIs extend beyond clinical outcomes and also encompass organizational, behavioral, and technical dimensions [7]. These characteristics also apply to DPHIs [11-13]. Consequently, the evaluation of DHIs as well as DPHIs is a methodological challenge [1,6,8,9], and existing established methods like health technology assessment (HTA), evidence-based medicine (EBM), and randomized controlled trials (RCTs) are limited in their application [16-18]. HTA is a process for the systematic evaluation of medical procedures and technologies, with a focus on assessing the health benefits and costs associated with the use of therapeutics, medical products, and diagnostic procedures [17]. Therefore, this methodology is not immediately appropriate in the context of the evaluation of DHIs [17,19]. EBM considers that decisions about the care of patients should be based on the best available external clinical evidence from systematic research in combination with the clinical expertise of health professionals. Regarding DHIs, there are more aspects to evaluate than clinical aspects, so it requires other distinct approaches than the usual suggested process to gather evidence within EBM [20]. Although RCTs are considered as the gold standard [6], especially in the context of evaluating DHIs, they are a much-discussed topic. Due to the complexity of DHIs, the applicability of RCTs is widely criticized [3,6,9,15,16,18]. A comprehensive evaluation of DHIs is essential for generating robust evidence [14,20], contributing to their long-term successful implementation and aiding in the realization of the full benefits of DHIs [15,20]. This principle also applies to DPHIs, which should adhere to an evidence- and needs-based approach, incorporating a participatory user-targeted development design to enhance acceptance of the intervention within the population [11,13,21].

The literature describes considerable variability and heterogeneity in how DHIs should be evaluated, which is attributable to the absence of a standardized, established, and broadly applicable approach for evaluating DHIs [3,6,15,20,22-25]. One possible explanation for the absence of a standardized and broadly applicable guideline for the evaluation of DHIs is that the evaluation of these technologies is complex and complicated by various fundamental issues [17]. Various European authorities and scientists have addressed this issue by developing and publishing proposed frameworks for the evaluation of DHIs, such as the Monitoring and Evaluation Guideline of the WHO, the Evidence Standards framework for digital health technologies of the National Institute for Health and Care Excellence (NICE), the report of the Expert Panel on effective ways of investing in health (EXPH), the Swiss Evaluation framework by Kowatsch et al, and the approach of Murray et al [1,5,14,17,24]. In this review, there is an orientation toward the WHO and NICE frameworks, for instance, in categorizing the evaluated DHIs and determining the evaluation criteria.

#### Status Quo of End-User Involvement in the Evaluation of DHIs

For various reasons, such as counteracting the nonuse of DHIs as well as DPHIs, evidence generation and evaluation should be practice-oriented, necessitating the integration of end users in this process [3,11,13,15,21,25-28]. In recent years, approaches, such as user-centered design (UCD), participatory health research (PHR), and public and patient involvement (PPI), have gained increasing importance in the health care sector, each contributing to the overarching goal of creating patient-centered, accessible, and equitable health care solutions.

UCD or human-centered design is rooted in human-system interactions and can be seen as a set of principles and strategies in the design and development of interactive digital health solutions, emphasizing the iterative research, design, and evaluation of services and systems by involving end users and stakeholders throughout the project life cycle [29-31]. PHR can be understood as a research paradigm rather than a research method, aiming to increase the participation of individuals whose life or work is the subject of research throughout the research process. The research process should be realized as a partnership among involved stakeholders (ie, with each other), instead of research on people as passive objects. Involved stakeholders could include academic researchers, health professionals, policymakers, and members of civil society [32]. INVOLVE, founded by the National Institute for Health Research (NIHR) and taken over by the NIHR Centre for Engagement and Dissemination in April 2020, defines PPI as research that is realized “with” or “by” members of the public, rather than “to,” “about,” or “for” them [33]. The term “public and patients” includes current, former, and potential patients; people who use health and social care services; people from organizations who represent other people using these services; and carers [34-36].

To sum up, regarding the definitional approaches of PHR and PPI, overlaps can be seen, especially regarding the statements that research should be performed with the research subjects rather than about them. UCD aligns with this principle but is more specifically focused on the design, development, and evaluation of interactive digital health solutions than on the scientific research context.

Through the involvement of end users in evaluation processes, acceptance and usability problems can be mitigated [15,26] and health interventions can be designed in a target group–specific and needs-based manner [27].

### Objectives

This scoping review has been conducted to understand and provide an overview of the current established processes for evaluating DHIs for public end users, such as citizens and patients. Previous reviews have focused on investigating which aspects of DHIs were evaluated during different development phases [20], investigating evaluation methods regarding specific criteria [28,37,38] or systems [39], investigating evaluation methods in specific medical contexts [22], investigating concrete evaluation methods despite RCTs [16], investigating economic evaluations of preventive DPHIs [12], or investigating general methods to evaluate the effects of DHIs for citizens by performing a review about reviews [6]. This review differs from those mentioned earlier as it provides a holistic overview of evaluation processes for DHIs for public end users, which is not limited to a specific medical field. The objectives are to capture the (1) evaluation methods and (2) evaluation criteria that are currently used to evaluate DHIs. Additionally, there is a focus on investigating the involvement of public end users in the further development of DHIs specifically developed for them. Therefore, the review addresses the following research questions (RQs):

1. Which research methods are used to evaluate DHIs for individuals or public end users?
2. Which evaluation criteria can be identified? Which evaluation criteria have been investigated?
3. In which way are individuals or public end users involved in the evaluation process?

In summary, this scoping review aims to provide an overview of the currently established processes for evaluating DHIs, including methods, indicators, and end-user involvement.

## Methods

### Overview

Through a scoping review, broad topics can be explored and gaps in the evidence can be identified [40,41]. Due to the nature of scoping reviews, we did not formally assess the risk of bias or methodological quality of the included studies [40,41]. This review was conducted based on the methodological framework for scoping reviews of Arksey & O’Malley [42] and adheres to the PRISMA-ScR (Preferred Reporting Items for Systematic Reviews and Meta-Analyses extension for Scoping Reviews) guidelines [43] incorporating the updates published by Peters et al [44] (Multimedia Appendix 1). The review protocol was registered a priori with the Center for Open Science (OSF) [45].

### Search Strategy

The primary information sources for this scoping review were scientific databases, namely PubMed, Scopus, and ScienceDirect. Additionally, Google Scholar and the reference lists of included papers were manually screened. The search was conducted in April 2023. The search string, developed iteratively by 3 domain experts, was equally applied across the abovementioned databases, and is detailed in Table 1. The search was carried out without the assistance of librarians. The main search terms “evaluation,” “digital health intervention,” and “user-centered” along with their synonyms were combined using Boolean operators (Table 1).

**Table 1:** Search string.

Operator	Field	Context	Focus
AND (main search term)	Evaluation (A)	Digital health intervention (B)	User-centered (C)
OR (synonyms)	evaluat*, evaluation method*, formative evaluation, summative evaluation, assess*	digital health intervention*, digital health technology, digital public health intervention*, digital health service*, electronic health record, mHealth, eHealth, health information technology, health Information platform, health diary	user-oriented, user-centered

The search string for each of the 3 databases is listed in Multimedia Appendix 2. The results of each database were stored in Citavi (Swiss Academic Software) and exported into Microsoft Excel files.

### Eligibility Criteria

Literature is eligible for inclusion if it describes evaluation methods or evaluation criteria for DHIs primarily aimed at public end users. Additionally, DHIs must be usable without the assistance of a health professional. The scoping review included all types of DHIs, including DPHIs without any exclusions, as long as the evaluation included an explorable version of the DHI. Moreover, literature that documents the integration of end users in the evaluation process is also eligible for inclusion. According to a previous manual search, it appears that the term DHI was more commonly used approximately 15 years ago. Additionally, the authors assumed that the introduction of the iPhone in 2007 led to an increase in mHealth evaluation studies. Therefore, literature published in the last 15 years (2008-2023) was included in the search. The eligibility criteria relevant to this scoping review are detailed in Textbox 1.

**Eligibility criteria.**

**Inclusion criteria**

- Targeted population: Primary end users of digital health interventions (DHIs) are individuals, such as patients and the public. The DHI can be used on its own. No limitations on the number of participants, their gender, or their origin.
- Study design: Original peer-reviewed studies, conference papers, book chapters, and grey literature, such as organizational reports.
- Context/field–DHI: DHIs can occur in the form of, for example, patient portals, platforms, web or mobile applications, or patient access to electronic health records. No exclusion of specific types of DHIs. There should be an explorable version of the DHI, which means at least a low-fidelity prototype, within the evaluation.
- Context/field–evaluation: The study proposes or describes the evaluation or assessment of a DHI. Iterative evaluations primarily used for requirement engineering or assessing basic needs are excluded or not focused.
- Context/field–end-user involvement: End users are kind of actively integrated into the evaluation process.
- Accessibility: Full text is freely available on the internet or after contacting the author.
- Language: English or German.
- Year: Published in the last 15 years (2008-2023).

**Exclusion criteria**

- Targeted population: End users are health professionals. The DHI can only be used with assistance from a health professional.
- Study design: Not peer-reviewed papers, preprints, reviews, comments, presentations, protocols, or posters.
- Context/field–DHI: There is no existing explorable version of the DHI.
- Context/field–evaluation: The paper describes a framework or a general overview of evaluation types and methods. The study only focuses on requirements of engineering processes, which means there is no explorable DHI (prototype or final DHI) available. Studies addressing only specific health issues designed to answer clinical research questions.
- Context/field–end-user involvement: The study did not include end users in the evaluation process. End users are only passive data objects, which means that they are not addressed with specific questions.
- Accessibility: Full text is not available on the internet or by contacting the author.
- Language: Other than English or German.
- Year: Literature older than 15 years.

### Process of Study Selection

The electronic search results were stored in Microsoft Excel. An initial selection based on language and publication year was partially conducted within the scientific databases. To select the search results, duplications were first removed. Subsequently, titles and abstracts were screened, and literature not meeting the eligibility criteria was excluded. To avoid reviewer bias, the screening process was partially conducted by different researchers. A random sample of 400 titles (400/2896, 13.8%) was additionally screened by 2 independent researchers. Furthermore, the entire abstract screening was conducted by 2 independent researchers. In case of ambiguities regarding eligibility, discrepancies were discussed until a consensus was reached. Finally, full texts were screened against the eligibility criteria.

### Data Extraction and Analysis

Data extraction was conducted to categorize the included papers for evidence synthesis. Data from the included sources were systematically extracted and organized into a predeveloped Excel spreadsheet. The data coding sheet was created within the research team and refined iteratively. The following data were extracted: bibliographic information (eg, author, year of publication, title, DOI), characteristics of the evaluated DHIs (eg, type of DHI classified according to the WHO and NICE, addressed medical issue, and intended use setting), evaluation methods (eg, study design, use of standardized approaches, amount of different methods, methods such as surveys or questionnaires, interviews, task or scenario completion, thinking aloud, system data analysis, free-testing phase with the duration, focus groups, and others), evaluation criteria (eg, aspect of assessment, explanation or definition of the evaluated criteria or indicators, evaluated indicators such as clinical outcomes, user behavior change, user experience, technical performance, content performance, actual system usage, suggestions for improvement, and others), and type of end-user involvement (eg, passive data object, active data object, and qualitative data subject). As part of this phase, classification schemes for the DHIs and the evaluated indicators were formulated using inductive category formation, whereby the extracted data served as the basis. This aimed to cluster all extracted DHIs and indicators within the context of this scoping review. The key terms used are outlined in Tables S1-S3 in Multimedia Appendix 3, with consideration of the wide array of DHIs as well as evaluated indicators along with the lack of a universally used classification framework.

## Results

### Overview

**Figure 1:**
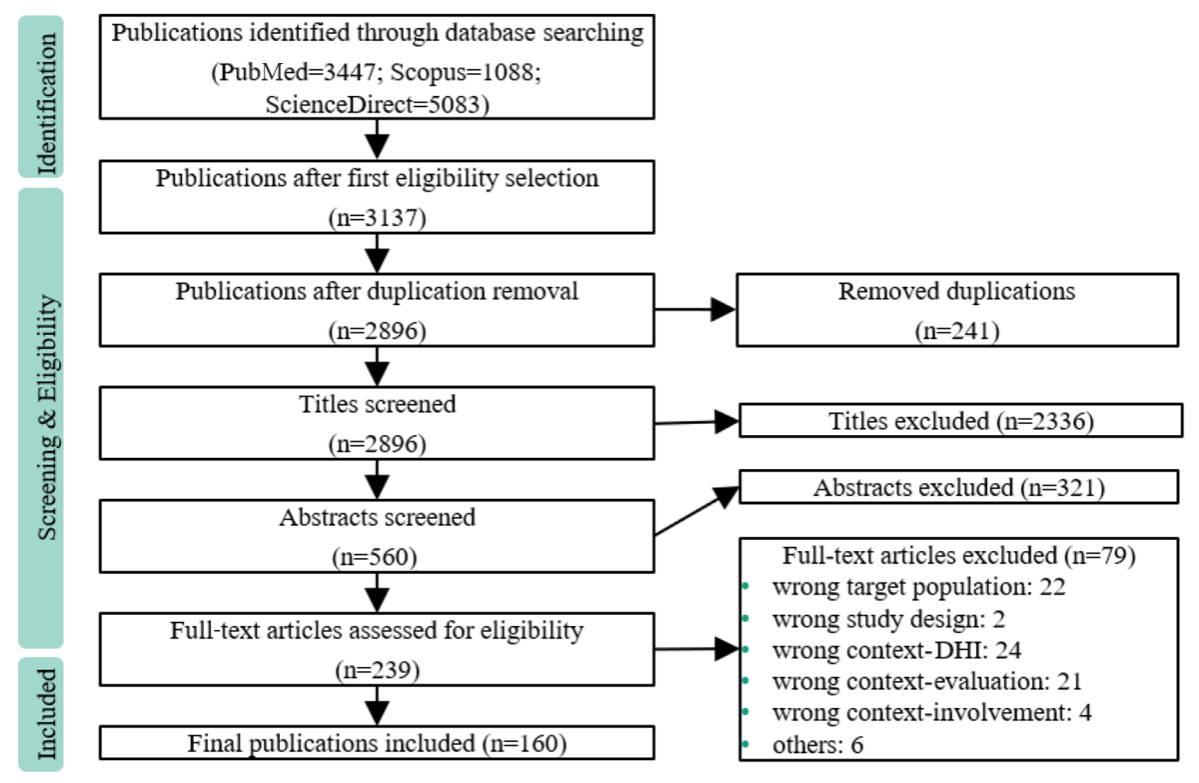
PRISMA (Preferred Reporting Items for Systematic Reviews and Meta-Analyses) flowchart. DHI: digital health intervention.

A total of 9618 articles were identified in April 2023 from the search strategy across the 3 scientific databases used. Through initial selection from the databases and the removal of duplicates, 2896 records remained for title screening. After this phase, 560 records remained for abstract screening. A total of 239 records were selected for full-text screening. During this stage, 79 records were excluded. Finally, 160 full-text articles met the eligibility criteria, resulting in 200 evaluations being derived and analyzed. This is explained by the observation that several papers (38/160, 23.8%) included multiple rounds of evaluation, with each employing different methods. For a brief overview of the included papers, 160 articles were relevant, whereas for analysis purposes, the number of evaluations resulting from the included papers was relevant. The data extraction chart is presented in Multimedia Appendix 4. The screening process is shown in Figure 1.

### Characteristics of the Included Studies and DHIs Examined in These Studies

The included studies were published between 2010 and 2023, and the number of published studies increased over the years. Specifically, 59 studies (59/160, 36.9%) were published before 2020, and 101 studies (101/160, 63.1%) were published from 2020 to April 2023.

In order to categorize the DHIs investigated in the articles, it was decided to use the WHO and NICE classification schemes [5,10]. There were many different kinds of DHIs, ranging from simple websites for information purposes to complex multimodal interventions. However, these established classification schemes reached their limits, as DHIs often serve multiple functions. As a result, more than a quarter of the DHIs (44/160, 27.5%) could not be clearly categorized within either scheme. Therefore, a summarizing classification scheme to be used in the context of the scoping review was formulated by using inductive category formation whereby the extracted data served as the basis (Table S1 in Multimedia Appendix 3). According to this scheme, most DHIs (42/160, 23.8%) supported the self-management of health and care, followed by DHIs that were used as a digital supportive component of a treatment (31/160, 19.4%).

In 66 articles (66/160, 41.2%), the primary focus was on the evaluation process. In 94 articles (94/160, 58.8%), the evaluation process was addressed secondarily, as part of a comprehensive description of the DHI development. Table 2 summarizes the characteristics of the included studies and the DHIs examined in these.

**Table 2:** Characteristics of the included articles.

Characteristic			Value (N=160), n (%)
**Year of publication**			
	2010-2011	2 (1.3)	
	2012-2013	4 (2.5)	
	2014-2015	5 (3.1)	
	2016-2017	17 (10.6)	
	2018-2019	31 (19.4)	
	2020-2021	52 (32.5)	
	2022-2023	49 (30.6)	
**Focus of the article**			
	Primary focus on evaluation	66 (41.2)	
	Secondary focus on evaluation	94 (58.8)	
**DHI^a^ categorization in established schemes**			
	Unclear or multiple	44 (27.5)	
	Categorized	116 (72.5)	
**DHI categorization in newly developed schemes**			
	Interaction with care provider: data transfer	2 (1.3)	
	Interaction with care provider: communication	5 (3.1)	
	Monitoring	22 (13.7)	
	Tailored information	22 (13.7)	
	Nontailored information	24 (15.0)	
	Digital supportive treatment component	31 (19.4)	
	Self-management	54 (33.8)	
**Addressed medical issues of DHIs**			
	Prevention or promotion	6 (3.8)	
	Generic	20 (12.5)	
	Mental	33 (20.6)	
	Somatic	101 (63.1)	
**Intended use setting of DHIs**			
	Nursing or retirement home setting	1 (0.6)	
	Rehabilitation setting	7 (4.4)	
	Clinical or stationary setting	8 (5.0)	
	Ambulant or primary care setting	40 (25.0)	
	Prevention or promotion	42 (26.2)	
	General health care setting	62 (38.8)	

^a^DHI: digital health intervention.

### Overview: Evaluation Methods

To answer RQ1 (“Which research methods are used to evaluate DHIs for individuals or public end users?”), the extracted data were analyzed to provide an overview of the used methods.

**Figure 2:**
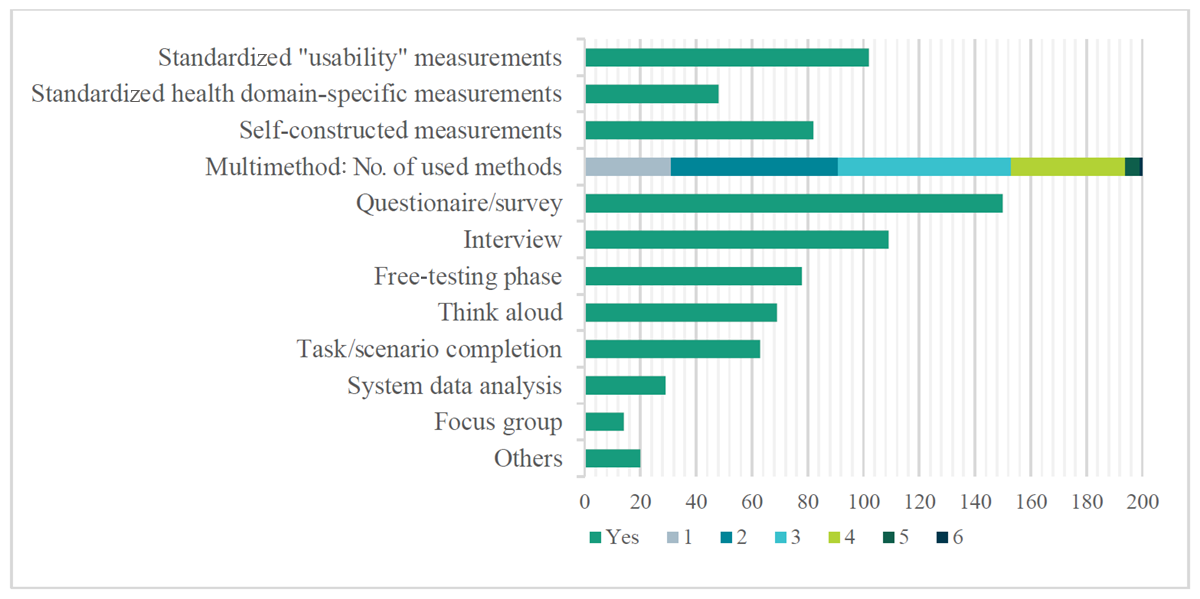
Research methods.

Analysis of the 200 evaluations revealed that a mixed-methods study design was most commonly used (121/200, 60.5%), followed by qualitative (42/200, 21.0%) and quantitative (37/200, 18.5%) study designs. Mostly, a combination of 2 (60/200, 30.0%) or 3 (62/200, 31.0%) different research methods was applied. Surveys were the most commonly used methods (150/200, 75.0%), followed by interviews (109/200, 54.5%) and testing phases (78/200, 39.0%). The duration of the testing phases predominantly exceeded 1 month (31/200, 15.5%), followed by durations between 1 week and 1 month (21/200, 10.5%) and those shorter than 1 day (13/200, 6.5%). Figure 2 provides a detailed overview of the specific research methods used.

As depicted in Figure 2, in over half of the evaluations (102/200, 51.0%), standardized usability scales, measurements, or questionnaires were used. Self-constructed measurements were used in 41.0% (82/200) of evaluations, while standardized health domain–specific measurements were used in 24.0% (48/200) of evaluations.

**Figure 3:**
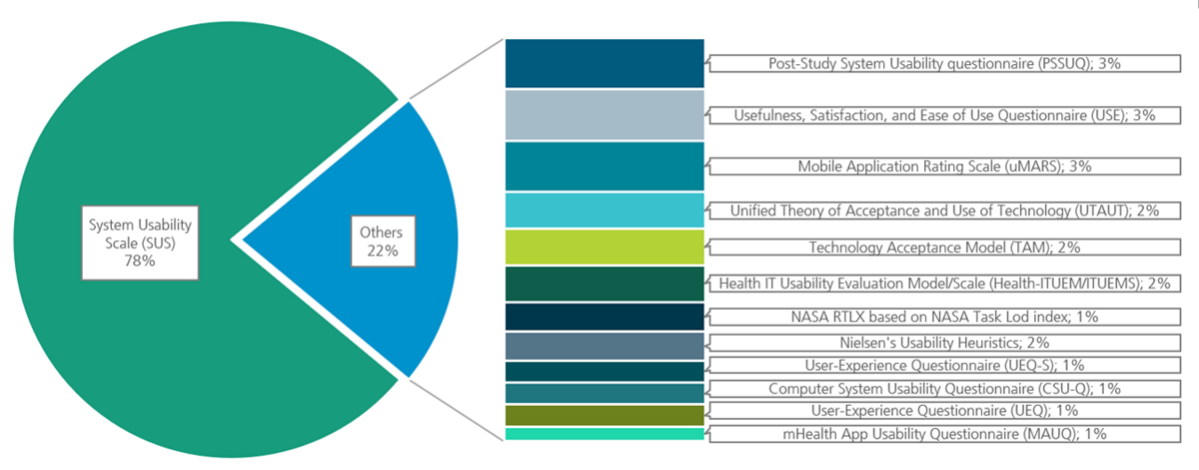
Overview of standardized usability measurements. For measurements that appeared in fewer than two evaluations, only health-specific ones have been listed.

To examine the commonly used standardized “usability” frameworks, Figure 3 visualizes most of the used measurements.

### Overview: Evaluation Criteria

To answer RQ2 (“Which evaluation criteria can be identified? Which evaluation criteria have been investigated?”), extracted data were analyzed to create a holistic picture of the used evaluation criteria.

**Figure 4:**
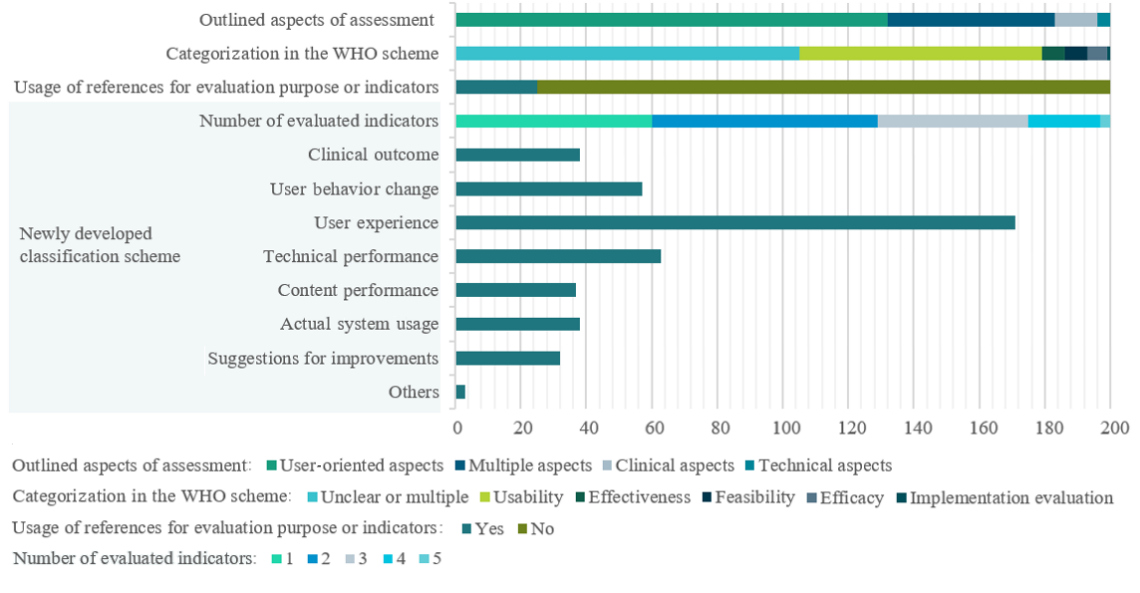
Research criteria.

Two-thirds (132/200, 66.0%) of the generally outlined aspects of assessments were user-oriented, followed by multiple aspects (51/200, 25.5%), clinical outcomes (13/200, 6.5%), and technical aspects (4/200, 2.0%). In order to categorize the evaluation criteria, it was intended to map the extracted evaluations into the established classification scheme of the WHO, which involves feasibility, usability, efficacy, effectiveness, and implementation research [14]. Similarly, when attempting to map evaluation criteria into the WHO classification scheme, limitations were encountered as there were multiple criteria mentioned by the authors or a classification was unclear because the terms were used differently. Moreover, the underlying descriptions or definitions of the terms were based on a variety of approaches and sources, and the used terms also varied. For example, in some evaluations, usability was defined and therefore measured by underlying indicators like effectiveness, efficiency, and satisfaction [46-49] or others like acceptance and feasibility [50,51]. In other cases, some of the previously listed underlying indicators were indicators of other criteria like feasibility [52-55] or were seen as independent criteria, for example, by the WHO or others [14,56-60]. Consequently, more than half of the evaluations (105/200, 52.5%) could not be clearly mapped to one of the WHO-described criteria, as shown in Figure 4.

To further explore term usage, the sources or references referred to by the authors of the included articles were examined. It was apparent that in 87.5% (175/200) of evaluations, no information or literature references were provided to guide the terms used. Out of the 25 evaluations (25/200, 12.5%) that referred to literature to describe the used terms, ISO 9241-11 was mostly referred (12/200, 6.0%), followed by multiple authors (9/200, 4.5%), Nielsen (3/200, 1.5%), and other references used once like the Hix & Hartson or “Fit between Individuals, Task, and Technology” (FITT) framework.

As an alternative to the WHO classification scheme, a new scheme was formulated for use in this scoping review. Inductive category formation was used to create the new scheme, whereby all indicators extracted from the evaluations served as the basis of the data. This process led to the consolidation of 8 criteria dimensions, which are described in Table S2 in Multimedia Appendix 3, along with their associated indicators. Figure 4 visualizes the described findings and the classification according to the newly developed scheme.

### Overview: End-User Involvement

To answer RQ3 (“In which way are individuals or public end users involved in the evaluation process?”), extracted data were analyzed to investigate end-user involvement in the evaluation of DHIs that are primarily used by them.

To investigate end-user involvement, the ways in which they were involved were categorized as follows: (1) passive data objects, where citizens or patients do not have an active role, such as in responding to surveys, and evidence is gathered, for example, by analyzing system data; (2) active data objects, characterized by consciously responding to surveys with predefined options or participating in task or scenario-based sessions; and (3) qualitative data objects, where patients or citizens provide individual responses, allowing them to express and explain their views and emotions. These groups are further described in Table S3 in Multimedia Appendix 3. The majority of evaluations actively involved public end users. In 83% (166/200) of evaluations, end users actively assessed the DHIs using predefined answer options, and in 73% (146/200), public end users and patients had the opportunity to individually express their views and emotions regarding the DHIs. The majority of evaluations referenced user-centered approaches (145/200, 72.5%), with UCD being the most commonly mentioned (133/200, 66.5%). Additionally, related approaches, such as participatory design, human-centered design, and co-design or co-creation, were mentioned alternatively or in combination with UCD. In 25.5% (55/200) of evaluations, none of these user-centered approaches were mentioned.

## Discussion

### Principal Findings

This scoping review provides a holistic overview of the way DHIs for public end users are evaluated. A total of 160 studies resulting in 200 evaluations were included in this review. It focused on assessing the range of scientific literature concerning various methods, indicators, and types of public involvement. Research in the field of DHI evaluation seems to be in its early stages. Although studies from the last 15 years were eligible, the oldest eligible study was from 2010, and until 2016, the number of studies was in the single-digit range. The majority of eligible publications were from 2020 onwards (101/160, 63.13%), indicating the growing relevance of evaluating DHIs for public end users. Similar findings are reported in other literature [3,4,6,28].

It has been shown that there is a broad range of different DHIs with complex functions, and thus, some cannot be clearly categorized in established schemes like those of the WHO or NICE. Furthermore, there is a lack of methodological consistency, and references to existing frameworks, such as the Monitoring and Evaluation Guideline of the WHO, the Evidence Standards framework for digital health technologies of the NICE, the report of the EXPH, the Swiss Evaluation framework by Kowatsch et al, and the approach of Murray et al [1,5,14,17,24], are notably absent. The bare use of standardized frameworks was also concluded by another review [20]. Instead of these, standardized usability measurements were used in more than half of the evaluations, although most of these measurement instruments are not specially developed for the health care context (eg, System Usability Scale [SUS]). A major use of standardized measurement methods also resulted from a review of a specific medical field [22]. In other cases, standard measurements were considered insufficient or found to be too complex [60], and thus, self-constructed surveys are often developed. On the one hand, this raises questions about differences regarding measurement quality, because some self-constructed measurements appear to not be validated. On the other hand, the use of different measurement methods (some validated and some not validated) complicates the comparability of the results. Similar results were found in the literature or in former reviews [28]. In addition, an important question is why current frameworks that are developed by credible institutions or scientists and explicitly focus on the health care sector are not being considered.

In terms of the evaluation criteria, similarities to previously described observations can be seen. As with methodology, no references to existing frameworks, such as the Monitoring and Evaluation Guideline of the WHO, the Evidence Standards framework for digital health technologies of the NICE, the report of the EXPH, the Swiss Evaluation framework by Kowatsch et al, and the approach of Murray et al [1,5,14,17,24], were mentioned. In the few evaluations that referred to existing literature to explain the used terms, ISO 9241-11 was mostly mentioned, although this has not been specially developed for the health care context. Regarding the mapping of the extracted large amount of evaluation criteria into the established classification scheme of the WHO, a similar circumstance to DHI mapping is noticeable. A clear categorization in established schemes is not possible because of a missing commonly agreed definition and therefore differing usage of the evaluated indicators. The lack of a uniform definition and the variability in term usage raise concerns about whether this impedes the sharing of evidence among eHealth interventions due to insufficient comparability of the results. The same thoughts have been reported in the literature [7,20].

The literature calls, for example, for UCD processes to increase the involvement of different end-user groups [3]. The results showed that the majority of evaluations referred to user-centered approaches and actively involved public end users in the evaluation process.

In summary, the analysis revealed that there is neither a consensus on the methods for evaluating DHIs nor a commonly agreed definition or usage of the evaluated indicators, resulting in a broad variety of evaluation practices. This aligns with the findings of the existing literature [23,61]. Although several frameworks exist, the problem of heterogenicity and variability according to the evaluation of DHIs remains, and these circumstances appear to hamper gathering of reliable evidence through evaluation.

To address these challenges, practical suggestions and implications for further research have been identified and will be described subsequently.

### Implications for Practice and Further Research

**Figure 5:**
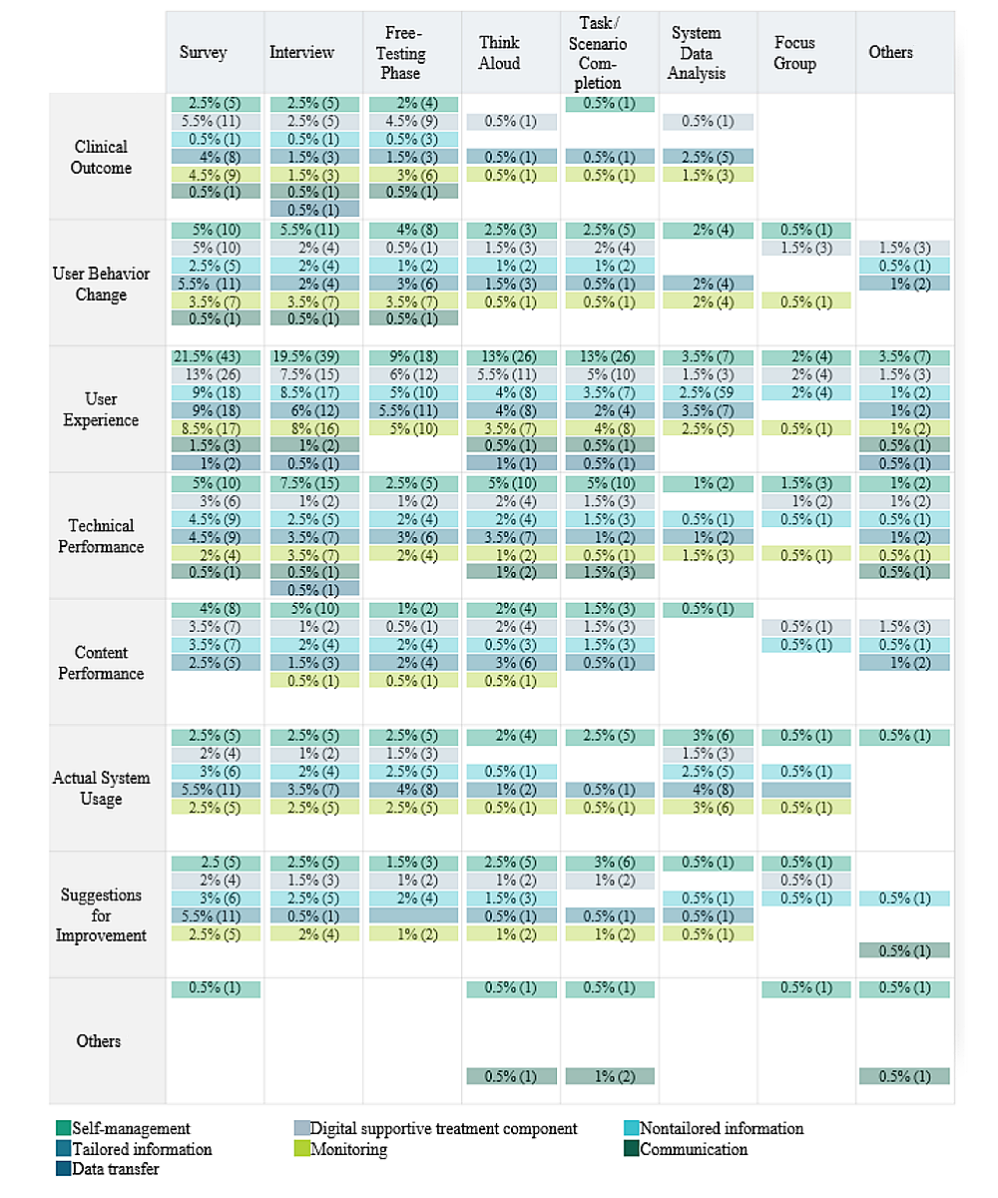
Results matrix method and criteria per digital health intervention. Others include cognitive walkthrough, observation, informal conversation, design workshop, and eye tracking.

To consolidate the previously mentioned results on evaluation methods and criteria for each DHI, according to the newly established categorization scheme, Figure 5 presents a results matrix. Considering the observation that established frameworks seem to not be referred, this figure is not intended to function as a framework. Its purpose is to assist developers, evaluators, researchers, and others in this field with the decision-making process by providing an overview of how DHIs have been evaluated by other people. This matrix can address the methodological challenge by providing initial practical guidance or decision-support for those developing and evaluating DHIs for public end users, offering a foundation for considering appropriate ways to evaluate their own DHIs.

As long as there is no commonly agreed and broadly applicable approach for evaluating DHIs, certain aspects derived from this review should be considered and described in evaluation studies to enhance the quality and comparability of the results, thereby creating evidence. Following the approach of Murray et al [1], a question-driven approach has been chosen for outlining these aspects.

#### What Type of DHI is at the Center of the Evaluation? What are its Main Characteristics and Intended Functions?

Describing the characteristics and key components of a DHI is crucial for comparability, and it forms the basis for selecting an appropriate methodology for the study. This description includes determining its primary functions, its user interface, the health domain it addresses, the specific medical issue it targets, the population it is designed for, and the intended use setting. This aligns with suggestions from existing frameworks [1,5,17]. Moreover, evaluators should reference established classification systems, such as those provided by the WHO or NICE [5,10]. This approach can help in creating a shared understanding and definition of the DHI context, as recommended in the literature [9].

#### What are the Reasons Behind Choosing the Research Method? What Roles do Established Measurement Methods Play?

While the description of the chosen methods is standard in research, the underlying reasons or decision-making processes for their selection are often not detailed. Therefore, it is essential that evaluators not only carefully select methods suitable for DHI evaluation but also provide clear justifications for these choices [17]. This transparency in decision-making enhances the reliability and validity of the research. Established measurement methods should be given due consideration in the evaluation process, and when these are applicable and relevant, they should be integrated into the study. Literature shows different reviews exploring potential frameworks that can be applied to DHIs [18,24]. If established methods are deemed inappropriate or inapplicable for the specific context of the DHI being evaluated, it is important to briefly explain the reasons for the exclusion. This approach not only adheres to high research standards but also helps in understanding the unique aspects of the DHI that necessitate a deviation from conventional methods.

#### Which Criteria are Being Evaluated? Which Established Sources or References are Considered Regarding the Evaluated Criteria?

The evaluated criteria and underlying measured indicators should be briefly described by considering established references. Clarity in defining these criteria is essential for creating a standardized and shared understanding of the evaluation objectives. The selection of the criteria should be informed by established references and frameworks in the field of digital health. A possible source to refer to for justification in the context of digitalization and health is the WHO, as it lists and defines criteria, such as feasibility, usability, efficacy, and effectiveness [14]. Another valuable reference is the Swiss Evaluation framework by Kowatsch et al [24], which outlines various criteria specific to DHIs. Referring to established references helps in grounding the evaluation with recognized standards and contributes to the creation of a shared language and understanding in the field, as suggested in the literature [9]. This approach can facilitate comparability across studies and contribute to the broader discourse on DHI efficacy and impact.

#### In Which Way are End Users Involved in the Evaluation Process?

In accordance with the NICE framework, it is important to detail how representatives from intended end-user groups are incorporated into the evaluation [5]. This should include specifying their roles and the extent of their participation, whereby approaches, such as PHR and PPI, could be referred. Given the potential agile nature of DHI development, these approaches could reach their limits, and referring to the principles, strategies, and methods, UCD can be highly effective in ensuring meaningful end-user involvement.

The following implication for further research can be derived from the scoping review. During the scoping review, the question arose of why current established measurements or frameworks, which are developed by credible institutions or scientists and explicitly focused on the health care sector, are not being referenced. This potential research-practice gap could be addressed by further research, for example, by investigating the discrepancies between scientific best practices in user-centered evaluation strategies and the suggestions from established frameworks within the processes of real-world user-centered evaluation. In addition, DHIs are developed in not only academic settings but also commercial sectors. As stated in the limitations, the results of evaluation studies are not always published, especially those from the commercial sector. Therefore, it could be interesting to investigate evaluation methods, particularly in this field. Similar thoughts are reported in the literature [28]. Regarding the involvement of public end users, it would be interesting to investigate from a public end-user perspective which methods are suitable for providing close-to-reality feedback. This involves questions, such as “How do they like to explore DHIs?” “Are the measurement methods currently used by evaluators suitable for conveying the benefits to public end users in a realistic way, thus enabling them to assess the value?” and “From their point of view, which approaches are tangible and can be transferred to the reality of care?” Furthermore, it could be interesting to develop and investigate a reporting guideline that ensures an evidence-based minimum set of items for reporting evaluation processes on DHIs. Considering the incorporation of potential end users, further research could focus on reporting guidelines consisting of standardized components regarding the participation or involvement of end users in the development or evaluation of DHIs.

### Limitations

This scoping review was conducted based on the methodological framework for scoping reviews by Arksey & O’Malley and complies with the PRISMA-ScR guidelines. To the best of our knowledge, the results provide the first holistic overview of scientific research on evaluation methods and indicators in the context of DHIs for public end users, as the scope is neither specific to medicine nor specific to criteria.

However, the review has some limitations. One limitation is associated with the way evaluation studies are published. Generally, evaluations of DHIs are published in one of the following two ways: different evaluations conducted during the development process of a DHI are published in separate papers or the entire development process is described in a single paper. In the latter case, there are often no detailed descriptions of the evaluation process, as it is not the main focus of the publication. This lack of detail impedes a comprehensive and thorough overview of the evaluation methods and indicators of DHIs throughout their development and actual use. Another limitation is that the results of evaluation studies, especially from the commercial sector, are not always published [20,23,28], which can hamper a comprehensive overview. Additionally, in line with the nature of scoping reviews, there is neither a quality assessment of the included studies and evaluations nor a quality assessment of the extracted methods, criteria, and modes of public involvement. These aspects are presented as reported by the authors of the primary studies.

### Conclusions

This scoping review provides a comprehensive overview of the current methods used in evaluating DHIs for public end users. The analysis revealed that there is neither a consensus on methods for evaluating DHIs nor a commonly agreed definition or usage of the evaluated indicators, resulting in a broad variety of evaluation practices. Although several frameworks exist, the problem of heterogenicity and variability according to the evaluation of DHIs remains, and these circumstances seem to hamper gathering reliable evidence through evaluation. Recommendations are derived from the findings in order to enhance the quality and comparability of the results of evaluation studies. It is important to note that the results are not intended to serve as a framework or as best-practice recommendations. Investigating these aspects could form a part of future research endeavors. We demonstrated that the research field is complex, heterogeneous, and broad, and our findings provide the first overview and have identified research gaps that could be addressed further. In conclusion, uniform use of terms, particularly regarding evaluation criteria and DHI classification, within the digital health sector could facilitate the transferability of results among similar evaluation studies. This standardization could significantly contribute to the cohesiveness and effectiveness of research in this evolving field.

## Appendix

Multimedia Appendix 1 PRISMA-ScR (Preferred Reporting Items for Systematic Reviews and Meta-Analyses extension for Scoping Reviews) checklist.

Multimedia Appendix 2 Search strategy for each database.

Multimedia Appendix 3 Key terms.

Multimedia Appendix 4 Data extraction chart.

## Abbreviations

DHI: digital health intervention
DPHI: digital public health intervention
EBM: evidence-based medicine
EXPH: Expert Panel on effective ways of investing in health
HTA: health technology assessment
mHealth: mobile health
NICE: National Institute for Health and Care Excellence
NIHR: National Institute for Health Research
PHR: participatory health research
PPI: public and patient involvement
PRISMA-ScR: Preferred Reporting Items for Systematic Reviews and Meta-Analyses extension for Scoping Reviews
RCT: randomized controlled trial
RQ: research question
UCD: user-centered design
WHO: World Health Organization
