# Supplementary material for: Methods, Indicators, and End-User Involvement in the Evaluation of Digital Health Interventions for the Public: Scoping Review
Source: J Med Internet Res. 2024 May 31;26:e55714. doi: 10.2196/55714 (PMC11179021; doi:10.2196/55714)
Supplement: Multimedia Appendix 2 [file jmir_v26i1e55714_app2.docx]

**Multimedia Appendix 2.** Search strategy for each database.

| Database | Search term | Results |
| --- | --- | --- |
| PubMed | (“evaluat*” OR “evaluation method*” OR “formative evaluation” OR “summative evaluation” OR “assess*”) AND (“digital health intervention*” OR “digital health technology” OR “digital public health intervention*” OR “digital health service*” OR “electronic health record” OR “mHealth” OR “eHealth” OR “health information technology” OR “health Information platform” OR “health diary”) AND (“User-oriented” OR “User-centered”) | 3447 |
| Scopus | (“evaluat*” OR “evaluation method*” OR “formative evaluation” OR “summative evaluation” OR “assess*”) AND (“digital health intervention*” OR “digital health technology” OR “digital public health intervention*” OR “digital health service*” OR “electronic health record” OR “mHealth” OR “eHealth” OR “health information technology” OR “health Information platform” OR “health diary”) AND (“User-oriented” OR “User-centered”) | 1088 |
| Science Direct | (“evaluat*” OR “evaluation method*” OR “formative evaluation” OR “summative evaluation” OR “assess*”) AND (“digital health intervention*” OR “digital health technology” OR “digital public health intervention*” OR “digital health service*” OR “electronic health record” OR “mHealth” OR “eHealth” OR “health information technology” OR “health Information platform” OR “health diary”) AND (“User-oriented” OR “User-centered”) | 5083 |
