# Supplementary material for: Methods, Indicators, and End-User Involvement in the Evaluation of Digital Health Interventions for the Public: Scoping Review
Source: J Med Internet Res. 2024 May 31;26:e55714. doi: 10.2196/55714 (PMC11179021; doi:10.2196/55714)
Supplement: Multimedia Appendix 3 [file jmir_v26i1e55714_app3.docx]

**Multimedia Appendix 3.** Key terms.

Table S1. Newly developed DHI classification system of DHIs for public end users

| **Term** | **Description in the context of this scoping review** |
| --- | --- |
| plain info without tailoring | A DHI that offers not tailored information, such as websites, generic apps for prevention or promotion, education support and generic health services. |
| individual info with tailoring | A DHI that offers not tailored/ individualized information, such as platforms, apps that promote a healthy lifestyle and specific health services |
| Interaction with care providers- communication | A DHI that supports the communication between an individual and health professionals or clinical institutions, such as apps that work as communication channels. |
| Interaction with care providers- data transfer | DHIs that help to transport data safely, such as applications or platforms that support data entry into a personally controlled health record. |
| Monitoring | A DHI that works as some kind of health and care diary, apps that track conditions or symptoms, and apps that monitor patient-reported outcomes (ePRO-System). |
| self-management (incl. decision aid, care management) | DHIs that support individual health and care management including decision aid and care management. Examples could be apps for decision support or care coordination. Most of these DHIs have multiple purposes like monitoring, reminder functions etc. |
| digital supportive component of a treatment | A DHI that actively addresses a medical issue and supports on-site treatment like DiGAs, Internet-based cognitive behaviour therapy/iCBT or other digital therapies. |

Table S2. Newly developed classification scheme for evaluation criteria categorization

| **Criteria** | **Description in context of this scoping review** | **Examples of extracted indicators** |
| --- | --- | --- |
| ClinicalOutcome | Clinical outcomes are about measurable changes in health like health improvements, QUALY, suicidal ratings | Health-Related Quality of Life (HRQoL); self-reported quality of life; impact on health; health-specific outcomes; initial clinical outcomes; clinical outcomes; clinical measurements; QUALY; medical outcomes; psychological outcomes; impact on quality of life; body weight; preliminary effects; effectiveness (completeness and correctness of medication lists); |
| Users Behaviour Change | Users Behaviour Change is about changes in terms of health behaviour, like adherence, physical activity, knowledge increase, changes in self-efficacy, and user engagement. | engagement; adherence; barriers to adherence; impact on knowledge; health care utilization; app influence; social influence; impact; patient Activation; (changes in) self-efficacy, continuity of care; physical activity; effects on usual process; willingness to use the technology; Intention to use; behavioural intention; real use; potential use |
| User Experience | User Experience is about satisfaction, usability, acceptability, enjoyment, and task succession. It can measure the end-users attitude and usage towards the DHI. | acceptability/acceptance; ease of use; perceived ease of use; engagement; satisfaction; impressions; usefulness; perceived usefulness; user-friendliness; helpfulness; barriers; facilitators; task complementation/completion; task success; task burden; task performance; task duration; user operation time; efficiency (time on task); performance; general feedback; general experience; general impression; (perceived) physical experience; learnability; user experience; Attitudes Toward Technology, effort expectancy; performance expectancy; efficiency & effectiveness by assessing task time and errors made; usability; discomfort; insecurity; user reactions |
| Content Quality | Content quality is about how well the given information are understood by the end-users and therefore achieves its goals. | Content; Content quality; Content clarity; information quality; clarity of information; Clarity; cultural relevance; comprehensibility; comprehension; trustworthiness; credibility; wording; ease of understanding |
| Technical Performance | Technical Performance/ system attributes are about the functions and the appearance of the DHI like design, and user interface. It's about how well can the end-users handle the DHI. | design; user interface design; functionality; aesthetics; navigation; technical performance; technical difficulties; system architecture; patient safety; privacy; layout; features; visual experience; structure; wording; haptic experience; customization; reported technical errors; user interactions |
| Actual System Usage | Actual Usage/ System Use like actual usage rates | Usage; Interaction; Portal Use; user frequencies; User Activities; System Use; analysis of system data (number of users, visits, and page views and the frequency and duration of use) |
| Suggestions for Improvements | It is about individual suggestions for improving the DHI, like positive as well as negative criticism. | (Suggestions for) Improvement; theme development; recommendations for future; open-enden qualitative feedback; |
| Others | Criteria that cannot be assigned to the previously mentioned | eye movement; |

Table S3. Categorization of end-user involvement

| **Term** | **Description in the context of this scoping review** |
| --- | --- |
| passive data objects | Patients or the public provide passive data. Quantitative data collection is used to derive findings on effectiveness or similar, such as in RCTs or usage data analyses. Patients or the public do not provide concrete (predefined or open) answers to specific questions (part of quantitative research).  In concrete, there are no questions like: What do you think of X? |
| active data object | Patients or the public actively answer questionnaires with predefined answer options on specific questions, as in SUS, UTAUT or specially developed questionnaires. Or they test DHIs in certain settings. (Part of quantitative & mixed-method research)  In concrete, there are questions such as: What do you think of X with response options? Has health or behaviour changed? |
| qualitative data subject | Patients or the public give individual answers to questions, like in semi-structured interviews or open-ended questions in surveys. By sharing their feelings and impressions, as in think-aloud approaches, they have space to express and explain their opinions. This includes suggestions for improvements (part of qualitative & mixed-method research). In concrete, there are questions like ‘Tell me how you feel?’ |
